# Outcomes of a newly established bariatric program in a hospital without an intensive care unit: a retrospective analysis of safety and effectiveness

**DOI:** 10.20452/wiitm.2025.17975

**Published:** 2025-08-05

**Authors:** Przemysław Sroczyński, Krzysztof Jędras, Grzegorz Dobkowski, Agata Gaździńska, Dariusz Tomaszewski, Robert Drozdowski, Michał Janik

**Affiliations:** General Surgery Department Military Institute of Aviation Medicine Warszawa Poland; Laboratory of Dietetics and Obesity Treatment, Department of Psychophysiological Measurements and Human Factor Research Military Institute of Aviation Medicine Warszawa Poland; Anesthesiology Department Military Institute of Aviation Medicine Warszawa Poland; Internal Medicine Department Military Institute of Aviation Medicine Warszawa Poland

**Keywords:** bariatric surgery, Clavien–Dindo classification, patient safety, Roux‑en‑Y gastric bypass, sleeve gastrectomy

## Abstract

**INTRODUCTION:**

Bariatric surgery effectively contributes to substantial weight loss and improvement of obesity‑related comorbidities. Establishing new bariatric programs, particularly in hospitals without intensive care units, requires tailored implementation strategies and careful resource planning to ensure both safety and efficacy.

**AIM:**

The aim of this study was to retrospectively assess the safety and efficacy outcomes of a newly implemented bariatric program the Military Institute of Aviation Medicine.

**MATERIALS AND METHODS:**

This retrospective study evaluated all bariatric surgeries (n = 267) performed between September 2021 and December 2024 at our hospital following the establishment of a comprehensive bariatric program. The program included surgeon training, multidisciplinary team formation, protocol development, and infrastructure modifications. Patient demographics, operative details, complications, weight loss outcomes, and resolution of comorbidities were analyzed.

**RESULT:**

Laparoscopic sleeve gastrectomy (LSG) was the predominant procedure (85.77%), followed by laparoscopic Roux‑en‑Y gastric bypass (LRYGB, 13.86%). Mean (SD) operative time was 145 (35) minutes for LRYGB and 77 (19) minutes for LSG. Overall, 93.1% of the patients had no complications, with the rate of serious complications requiring surgical intervention (Clavien–Dindo grade IIIB) decreasing from 7.69% in 2021 to 0.97% in 2024. No mortalities occurred. Among the 112 patients (68.3%) with available follow‑up data, mean (SD) percentage of total weight loss was 29.61% (12.08%) for LRYGB and 25.92% (10.57%) for LSG. High rates of comorbidity resolution were observed for type 2 diabetes mellitus (90.91%), hypertension (65.79%), dyslipidemia (70%), and metabolic syndrome (91.67%).

**CONCLUSION:**

With careful planning, comprehensive team training, and adherence to established protocols, a new bariatric surgery program can achieve excellent safety and effectiveness outcomes from inception. The results validate our approach to program development and patient care.

## INTRODUCTION

Obesity has emerged as a critical public health issue worldwide, contributing significantly to morbidity and mortality.^1^;^2^ It is associated with comorbidities, including type 2 diabetes mellitus (T2DM), cardiovascular diseases, and various forms of cancer, necessitating effective interventions for its management. Bariatric surgery has been recognized as one of the most effective procedures for achieving sustainable weight loss, improving obesity-related comorbidities, and improving overall quality of life.^1^;^3^;^4^ Implementing new bariatric programs, especially in hospitals without intensive care units (ICUs), requires specialized strategies and meticulous resource planning to ensure safety and efficacy. This study examined a newly established bariatric program at a non-ICU hospital, addressing the literature gap regarding outcomes of such early-stage initiatives in limited-resource settings.

## AIM

The primary objective of this study was to retrospectively assess the safety and efficacy outcomes of a newly implemented bariatric program at our hospital. This involved evaluating the incidence of surgical complications and overall patient safety metrics. By analyzing these factors, we aimed to determine the program effectiveness and identify areas for potential improvement.

## MATERIALS AND METHODS

This retrospective study evaluated the safety and efficacy of the bariatric program initiated at the Military Institute of Aviation Medicine in Warsaw, Poland, in September 2021. The study period spanned from September 2021 through December 2024. We adhered to the Strengthening the Reporting of Observational Studies in Epidemiology guidelines for retrospective studies, ensuring rigorous and transparent reporting.^5^

The bariatric program was established in 2021, when the lead surgeon joined our hospital. Based on experience from high-volume bariatric centers, we developed a comprehensive framework defining program scope, eligibility criteria, and care protocols. We assembled a multidisciplinary team of surgeons, nurses, dietitians, and psychologists, and provided specialized training for all staff members involved in bariatric care.

The implementation of the bariatric program required significant structural and procedural changes. Our hospital lacked an established bariatric care team and access to an ICU. To address this, we developed an implementation strategy that involved: 1) phased introduction of procedures starting with sleeve gastrectomy (SG); 2) strategic scheduling of surgeries on Mondays and Tuesdays to ensure full postoperative support; 3) staff training across surgical, anesthetic, nursing, nutritional, and psychological domains; and 4) development of protocols for perioperative care based on guidelines.^1^;^6^;^7^;^8^ These adaptations allowed for safe initiation of the program despite infrastructural limitations.

We conducted a retrospective analysis of all bariatric surgeries (n = 267) performed at our hospital from September 2021 to December 2024. The eligibility criteria for bariatric surgery followed international guidelines^9^, including body mass index (BMI) thresholds and presence of obesity-related comorbidities. Data were extracted from patient medical charts, which provided comprehensive details on baseline characteristics, types of surgeries performed, and procedural specifics. The collected data included: patient demographics (age, sex, BMI, and comorbidities), type of bariatric procedure performed, operative time, postoperative outcomes (length of hospital stay [LOS] and postoperative complications according to the Clavien–Dindo classification).

Primary outcomes included operative safety measures (operative time, LOS, and complications classified according to the Clavien–Dindo system) and efficacy measures (percentage of total weight loss [%TWL] and resolution or improvement of obesity-related comorbidities).

We collected data regarding %TWL and changes in obesity-related comorbidities, including T2DM, hypertension, obstructive sleep apnea, dyslipidemia, and gastroesophageal reflux disease (GERD; requiring medications) at various follow-up intervals. In this study, the improvement of comorbidities was defined as either a reduction in medication dosage or complete discontinuation of medications used to treat a specific condition, as reported by the patients. Data on these effectiveness outcomes were collected through structured telephone interviews with the patients during follow-up.

### Statistical analysis

For continuous variables such as age, BMI, operative time, LOS, and %TWL, we calculated means (SDs), medians, and ranges. To compare the outcomes between different surgical procedures, nonparametric tests were employed, including the Wilcoxon test for analyzing differences in weight loss outcomes at 1-year follow-up. For categorical outcomes, the Fisher exact test was used. For all statistical analyses, a *P* value below 0.05 was considered significant. All analyses were performed using SAS OnDemand (SAS Institute Inc., Cary, North Carolina, United States).

### Ethics

Ethical approval for this study was granted by the institutional Ethics Committee (no number was assigned). Due to the retrospective nature of the study, informed consent was waived, but all patient data were de-identified to maintain confidentiality.

## RESULTS

The bariatric surgery program was successfully implemented in September 2021, with the first procedures performed the same month. By December 2024, a total of 267 bariatric procedures had been performed, indicating successful program adoption and implementation.

Three main bariatric procedures were performed during the study period: laparoscopic SG (LSG), laparoscopic Roux-en-Y gastric bypass (LRYGB), and single anastomosis duodeno-ileal bypass with sleeve gastrectomy (SADI-S). In 6 out of 267 patients, the adjustable gastric band was removed; however, these patients were not included in the final analysis. LSG was the predominant procedure, accounting for 85.77% (n = 229) of all surgeries, followed by LRYGB (13.86%; n = 37), and SADI-S (0.37%; n = 1; Table 1)

**Table 1:** Distribution of bariatric procedures by year

Procedure type	2021	2022	2023	2024	Total
LRYGB	5 (38.46)	6 (10.17)	13 (15.12)	13 (12.62)	37 (14.18)
LSG	8 (61.54)	53 (89.83)	73 (84.88)	89 (86.41)	223 (85.44)
SADI‑S				1 (0.97)	1 (0.38)
Total	13 (4.98)	59 (22.61)	86 (32.95)	103 (39.46)	261 (100)

Data are presented as numbers (percentages).

Abbreviations: LRYGB, laparoscopic Roux‑en‑Y gastric bypass; LSG, laparoscopic sleeve gastrectomy; SADI‑S, single anastomosis duodeno‑ileal bypass with sleeve gastrectomy

The patient population consisted predominantly of women (83.9%; n = 224), whereas men constituted 16.1% of the study group (n = 43). Mean (SD) age of the patients undergoing LRYGB was 42.19 (7.13) years (range, 31–57 y), while for the LSG patients, it was 38.58 (9.86) years (range, 20–68 y; *P* = 0.01). The SADI-S patient was 38 years old.

Mean (SD) preoperative BMI was 37.18 (5.19) kg/m² for the LRYGB patients and 41.28 (5.28) kg/m² for the LSG patients. The BMI of the individual undergoing SADI-S was 43.53 kg/m² (*P* <⁠0.001). These values are consistent with international guidelines^9^ for bariatric surgery eligibility.

Regarding obesity-related comorbidities at baseline, we observed the following in the follow-up cohort (n = 112): T2DM in 10% of the patients (exclusively in the LSG group; *P* = 0.36); hypertension in 34.82% of the participants (26.67% in the LRYGB and 36.08% in the LSG groups, respectively; *P *= 0.57); dyslipidemia in 8.93% of the patients (6.67% in the LRYGB and 9.28% in the LSG groups, respectively; *P* >0.99); and metabolic syndrome in 21.43% of the individuals (13.33% in the LRYGB and 22.68% in the LSG groups, respectively; *P *= 0.52).

The operative time varied considerably between the procedures, reflecting their different technical complexity. The LRYGB procedures had a mean (SD) operative time of 145 (35) minutes (range, 89–231 min), while the LSG procedures were completed in a mean (SD) time of 77 (19) minutes (range, 40–138 min; *P* = 0.04). The only SADI-S procedure was carried out in 273 minutes.

When analyzed by year, we observed a decreasing trend in mean operative time for the LRYGB procedures. Although this trend suggests improved surgical efficiency over time, the differences were insignificant. Details regarding operative times for each surgery type according to year are presented in Table 2.

**Table 2:** Operative time and length of stay by procedure type and year

Parameter	2021	2022	2023	2024	Overall	*P* value
Operative time, min						
LRYGB	172	145	141.85	139.15	145.49	0.35
	(17.89)	(25.69)	(37.39)	(40.58)	(35.48)	
LSG	63.75	84.6	81.14	70.92	77.36	<0.0001
	(16.2)	(17.91)	(16.99)	(20.05)	(19.36)	
Length of hospital stay, d						
LRYGB	3.4	3.83	3.77	3.46	3.62	0.69
	(0.55)	(1.6)	(0.73)	(0.66)	(0.86)	
LSG	3.5	3.25	3.43	3.42	3.38	0.52
	(0.53)	(0.59)	(0.83)	(0.86)	(0.78)	

Data are presented as mean (SD). Abbreviations: see Table 1

Mean (SD) LOS was 3.62 (0.86) days (median range, 3–7 d) for the LRYGB patients and 3.38 (0.78) days (range, 2–9 d) for the LSG patients, indicating a slightly shorter hospitalization period for the LSG procedures (*P* = 0.04). The SADI-S patient had a hospital stay of 4 days. The LOS remained relatively stable across the years for both procedures, with no differences observed for either the LRYGB or LSG groups (*P* = 0.77; *P* = 0.52, respectively; Table 2).

Overall, 93.1% of the patients had no complications (Clavien–Dindo grade 0), 4.6% experienced minor complications (Clavien–Dindo grade I), 0.38% had grade II complications, and 1.92% had more severe complications requiring surgical intervention (Clavien–Dindo grade IIIB). The distribution of complications was similar between the procedures, with 94.59% of the LRYGB patients and 92.83% of the LSG participants experiencing no complications. The analysis of complication rates by year showed consistent safety outcomes with no differences (*P* = 0.72). No complications were recorded for 92.31% of the patients in 2021, 91.53% in 2022, 93.02% in 2023, and 94.17% in 2024. Of special significance is the marked reduction in the number of Clavien–Dindo grade IIIB complications—those requiring surgical intervention—over the course of the program. The rates of these serious complications decreased from 7.69% in 2021 to 0.97% in 2024. However, the difference was not significant; Table 3).

**Table 3:** Postoperative complications by year (Clavien–Dindo classification)

Clavien–Dindo grade	2021	2022	2023	2024	Total
Grade 0	12 (92.31)	54 (91.53)	80 (93.02)	97 (94.17)	243 (93.1)
Grade I		3 (5.08)	5 (5.81)	4 (3.88)	12 (4.6)
Grade II				1 (0.97)	1 (0.38)
Grade IIIB	1 (7.69)	2 (3.39)	1 (1.16)	1 (0.97)	5 (1.92)
Total	13 (100)	59 (100)	86 (100)	103 (100)	261 (100)

Data are presented as numbers (percentages).

There were no mortalities in our series, further confirming the safety of the program.

Out of the 164 patients who underwent bariatric surgery in our center between 2021 and 2023, follow-up data regarding weight loss and comorbidity resolution were available for 112 patients, representing a 68.3% follow-up rate. The remaining 52 patients (31.7%) were lost to follow-up or did not complete the telephone survey. Among those with follow-up data, 57 patients (50.89%) provided 1-year data, 46 patients (41.07%) provided 2-year data, and 9 patients (8.04%) provided 3-year data.

When comparing weight loss outcomes between the 2 procedures, the patients who underwent LRYGB achieved considerably higher overall %TWL (mean [SD], 29.61% [12.08%]; n = 15) than the LSG patients (mean [SD], 25.92% [10.57%]; n = 97; *P* = 0.04). Across specific follow-up points, %TWL was greater after LRYGB than after LSG at 1 and 3 years, while at 2 years, the outcomes were comparable; none of these differences reached significance Table 4. These results demonstrate notable and sustained weight loss for both procedures over the 3-year follow-up, with LRYGB showing superior overall weight loss outcomes.

**Table 4:** Weight loss outcomes by procedure type and follow-up period

Procedure type	n	%TWL	*P* value
Overall weight loss			
LRYGB	15	29.61 (12.08)	0.04
LSG	97	25.92 (10.57)	
1-year follow-up			
LRYGB	7	29.9 (12.26)	0.11
LSG	49	24.31 (11.54)	
2-year follow-up			
LRYGB	4	26.46 (12.38)	0.52
LSG	42	27.57 (9.68)	
3-year follow-up			
LRYGB	4	33.1 (13.9)	0.3
Overall weight loss	Overall weight loss	Overall weight loss	

Data are presented as mean (SD).

Abbreviations: %TWL, percentage of total weight loss; others, see Table 1

Our telephone survey assessed the resolution or improvement of obesity-related comorbidities following bariatric surgery. For the patients with T2DM, we observed a remission rate of 90.91% (10 out of 11 individuals), with all cases of remission occurring in the LSG group. Hypertension resolved in 65.79% of the affected patients (25 out of 38), with a higher remission rate in the LRYGB group (75%) than the LSG patients (64.71%). Among the patients with preoperative dyslipidemia, 70% experienced complete remission (7 out of 10 individuals), including 100% remission in the single LRYGB patient with this condition and 66.67% in the LSG group.

Postoperative use of GERD medications was reported in 26.79% of the patients in the follow-up cohort. The analysis by procedure type showed a notable difference in the need for GERD medications after surgery: 20% of the individuals who underwent LRYGB required GERD medications, as compared with 27.84% of those who underwent LSG (*P* = 0.52).

## DISCUSSION

The retrospective analysis of our newly implemented bariatric surgery program demonstrates that with strategic planning, multidisciplinary team development, and strict protocol adherence, a safe and effective bariatric program can be successfully established at a hospital without prior experience in this specialty. Our general hospital setting without an ICU necessitated specialized planning, comprehensive staff training, and tailored scheduling adaptations—insights valuable for similar resource-limited institutions. The findings underscore several key aspects of program performance and patient outcomes deserving further examination.

The successful implementation of our bariatric program in September 2021 and the completion of 267 procedures by December 2024 indicate a robust adoption of bariatric surgery at our institution. This surgical volume, achieved within approximately 3 years, reflects effective community outreach, appropriate patient selection, and successful integration of bariatric surgery into the services offered by our hospital. While this volume may be modest compared with established high-volume centers, it represents a significant achievement for a newly developed program and aligns with recommendations that new programs gradually increase their surgical volume to ensure quality and safety.^10^;^11^

The predominance of LSG in our series (85.77%) reflects current global trends in bariatric surgery, where LSG has become the most frequently performed bariatric procedure due to its technical simplicity, favorable safety profile, and good weight-loss outcomes.^11^;^12^;^13^;^14^ The lower proportion of LRYGB procedures (13.86%) may reflect our cautious approach during the initial phase of the program, as LRYGB is technically more demanding and associated with a steeper learning curve. However, the proportion of LRYGB in our study was still higher than the national average.^11^

The observed reduction in operative time for the LRYGB procedures from 2021 to 2024, though not significant, suggests a positive learning curve effect. This improvement in surgical efficiency without compromising safety is an important marker of program maturation and surgeon skill development. The fluctuations in LSG operative times across the years, with initial increase followed by decrease, may reflect changes in case complexity, variations in teaching involvement, or modifications in surgical technique.^15^

Perhaps the most encouraging aspect of our results is the excellent safety profile of the program. With 93.1% of the patients experiencing no complications (Clavien–Dindo grade 0) and only 1.92% requiring surgical reintervention (Clavien–Dindo grade IIIB), our complication rates compare favorably with the established standards in bariatric surgery. A particularly noteworthy finding is the steady improvement in the safety profile over time, as evidenced by the progressive decrease in complications according to the Clavien–Dindo classification across the successive years. While 92.31% of the patients operated in 2021 experienced no complications, these rates reached 91.53% in 2022, 93.02% in 2023, and 94.17% in 2024. A nearly 8-fold reduction in complications requiring surgical reintervention demonstrates a substantial improvement in patient safety outcomes as the program matured. While the overall improvement in complication rates was not significant (*P* = 0.72), the consistent and substantial reduction in the most serious complications (Clavien–Dindo grade IIIB) represents a clinically meaningful advancement in the program safety profile. This progressive reduction in complications, particularly those requiring surgical intervention, reflects the benefits of accumulated team experience, refinement of surgical techniques, standardization of perioperative protocols, and continuous quality improvement processes implemented throughout the program’s development. The absence of mortality further validates its safety.

The safety results are particularly significant in the context of the ongoing debate about the necessity of having an ICU in hospitals where bariatric surgeries are performed. While this requirement is included in the Polish standards,^16^ a study by ^17^ shows that as many as 17% of bariatric centers do not have access to an ICU. Our results demonstrate that bariatric surgeries, with the maintenance of appropriate safety thresholds, can be safely performed in hospitals without an ICU.

The experience presented in this study may be particularly relevant to regional hospitals or developing centers where ICU access is limited or unavailable. Our phased, multidisciplinary approach—combined with adherence to evidence-based protocols—enabled us to maintain safety and achieve favorable outcomes. The findings support the feasibility of implementing bariatric programs outside of tertiary academic centers and may inform broader policy discussions about decentralizing access to metabolic surgery.

Mean (SD) LOS (3.62 [0.8] d for the LRYGB and 3.38 [0.78] d for the LSG procedures) appears slightly longer than that indicated in the reports from some high-volume centers in the United States, where same-day discharge or 1–2-day stays are increasingly common.^10^ However, our results align well with European practices and reflect our cautious approach during the program’s initial phase. As the program matures and enhanced recovery protocols are further refined, we anticipate a gradual reduction in hospital stay duration.

The weight loss results observed in our cohort (mean [SD] %TWL of 29.61% [12.08%] for the LRYGB and 25.92% [10.57%] for the LSG procedures) are consistent with published literature and indicate successful achievement of primary bariatric surgery goals. The slightly higher weight loss observed with LRYGB, as compared with LSG, though not significant in our analysis, aligns with larger studies that have demonstrated marginally superior weight loss with LRYGB.^18^;^19^ The sustained weight loss over the 3-year follow-up is particularly encouraging and suggests patient adherence to lifestyle modifications. The slight increase in %TWL from year 2 to year 3 in the LRYGB group is noteworthy and may reflect the durability of this procedure, though the small sample size at this time point (n = 4) limits definitive conclusions.

The impressive rates of comorbidity resolution observed in our patients underline significant health benefits extending beyond weight loss. The 90.91% remission rate in T2DM is particularly remarkable and consistent with the transformative metabolic effects of bariatric surgery documented in landmark studies.^20^;^21^ The high resolution rates for hypertension (65.79%), dyslipidemia (70%), and metabolic syndrome (91.67%) further validate the metabolic benefits of bariatric surgery and its potential role in reducing cardiovascular risk. The numerically higher rates of comorbidity align with previous research suggesting potentially superior metabolic effects with bypass procedures, though our subgroup sample sizes were too small for generalized conclusions.^18^;^19^

In our analysis of postoperative GERD management, a particularly notable finding was that 20% of the LRYGB patients still required proton pump inhibitor therapy despite undergoing what is traditionally considered an antireflux procedure. While this rate was lower than in the LSG patients (27.84%), the persistence of GERD medication needs in 20% of the LRYGB patients challenges the conventional expectation that this procedure universally resolves reflux symptoms. This suggests more complex mechanisms of post-LRYGB reflux than typically acknowledged, potentially involving altered esophageal motility or persistent esophageal hypersensitivity. Our findings indicate that while LRYGB may offer some advantages for GERD management, patients should be counseled that ongoing medication requirements remain a possibility even after this procedure.

Our study has several limitations that should be acknowledged. First, the retrospective design limits causal inferences and may introduce selection bias. Second, the 68.3% follow-up rate, while reasonable for a real-world setting, means that outcomes for approximately one-third of eligible patients remain unknown, potentially affecting the accuracy of our effectiveness estimates. Third, the assessment of comorbidity resolution relied on patient self-reporting during telephone interviews rather than standardized clinical measurements, which may limit precision.

Moving forward, our program aims to improve follow-up rates through more robust patient tracking systems and enhanced postoperative engagement strategies. Additionally, as our program matures, we intend to expand our offer of procedures to include revisional surgery and potentially SADI-S for selected patients with more severe obesity or specific metabolic indications.^22^;^23^

## CONCLUSIONS

The successful implementation of our bariatric program demonstrates that, with careful planning, structured team training, and strict adherence to established protocols, new bariatric surgery programs can achieve high standards of safety and effectiveness from the outset. The low rate of complications, substantial weight loss, and high resolution of obesity-related comorbidities observed in our cohort confirm the clinical soundness of our approach. Importantly, our experience shows that bariatric surgery can be safely introduced even in hospitals without access to an ICU, provided that a phased introduction strategy, tailored workflows, and continuous quality monitoring are in place. These findings may serve as a practical model for other institutions seeking to establish bariatric services in resource-constrained settings.
